# Comparative Evaluation of Antimicrobial Activity of Dentin Disinfection Protocols Against MRSA and *Candida albicans*: An In Vitro Experimental Study

**DOI:** 10.3390/jfb17090431

**Published:** 2026-08-27

**Authors:** Kinga Mária Jánosi, Diana Cerghizan, Izabella Éva Mureșan, Aurița Ioana Albu, Liana Claudia Dobreci, Liana Georgiana Hănțoiu, Alpár Kovács, Sharukh S. Khajotia, Cristina Nicoleta Ciurea

**Affiliations:** 1 Faculty of Dental Medicine, George Emil Palade University of Medicine, Pharmacy, Science, and Technology of Târgu Mureș, 38 Gh. Marinescu Str., 540139 Târgu Mureș, Romania; kinga.janosi@umfst.ro (K.M.J.); izabella-eva.muresan@umfst.ro (I.É.M.); ioana.albu@umfst.ro (A.I.A.); claudia.dobreci@umfst.ro (L.C.D.); liana.hantoiu@umfst.ro (L.G.H.); 2 Private Practice, 540501 Târgu Mureș, Romania; alparko@yahoo.com; 3 Division of Dental Biomaterials, Department of Restorative Sciences, College of Dentistry, University of Oklahoma Health Campus, Oklahoma City, OK 73117, USA; sharukh-khajotia@ou.edu; 4 Department of Microbiology, George Emil Palade University of Medicine, Pharmacy, Science, and Technology of Târgu Mureș, 540139 Târgu Mureș, Romania; cristina.ciurea@umfst.ro

**Keywords:** dentin disinfection, antimicrobial activity, chlorhexidine, hydrogen peroxide, photodynamic therapy, MRSA, *Candida albicans*

## Abstract

**Background:** Dentin disinfection plays an important role in prosthodontics, reducing the microbial load, preventing postoperative complications and enhancing the bond strength. This in vitro study aims to evaluate the antimicrobial efficacy of different dentin disinfection protocols, depending on microorganism type, application time, and dentin depth. **Methods:** A total of 144 extracted human molars were included in the final analysis. The exposed dentin surfaces were contaminated with methicillin-resistant *Staphylococcus aureus* (MRSA) or *Candida albicans (C. albicans)*. The specimens were disinfected using 2% chlorhexidine digluconate, 3% hydrogen peroxide, or photodynamic therapy with 1% toluidine blue. Microbial growth at superficial and deep dentin was assessed using semi-quantitative visual colony-density scores, resulting in 288 microbiological measurements. Ordinal generalized estimating equation (GEE) models, accounting for within-tooth correlation, were used to evaluate the factors associated with microbial scores. **Results:** The results showed statistically significant differences among protocols for MRSA (Wald χ^2^ = 62.98; df = 5; *p* < 0.001) and for *C. albicans* (Wald χ^2^ = 18.77; df = 5; *p* = 0.002). Specifically, 2% CHX applied for 30 s was associated with the greatest reduction in MRSA microbial scores (OR = 0.121; *p* = 0.009), whereas 3% H_2_O_2_ applied for 60 s was more effective against *C. albicans* (OR = 0.054; *p* < 0.001). PDT showed lower and less consistent antimicrobial effects under the tested parameters. **Conclusions:** The effectiveness of dentin disinfection depends on the microorganism, application time, and dentin depth. Complete elimination of the microbial load cannot be achieved using the disinfectants investigated.

## 1. Introduction

Dentin disinfection in prepared teeth is essential to prevent postoperative complications following prosthodontic treatments. In addition to their antimicrobial activity, disinfectants may influence the performance of resin cements used for cementing restorations. The tubular structure of dentin allows microorganisms to penetrate deeply, making their elimination challenging through disinfection before cementation [1].

The persistence of microorganisms within dentinal tubules after disinfection may compromise the long-term success of restorations [2,3]. Disinfection of the abutment surface aims to reduce the microbial load and prevent biofilm formation at the restoration–dentin interface. Conventional and modern dentin disinfection methods have been proposed in the literature. Chlorhexidine digluconate (CHX) and hydrogen peroxide (H_2_O_2_) are commonly used disinfectants in vital abutments [2,4,5,6]. The broad antimicrobial spectrum of these disinfectants has made their use popular in daily practice [2,7]. CHX is one of the most extensively investigated antimicrobial agents for dentin pretreatment owing to its broad-spectrum antimicrobial activity and its potential to inhibit dentinal matrix metalloproteinases and thereby preserve the resin–dentin interface [8]. However, contemporary evidence indicates that these laboratory-observed effects do not necessarily translate into a consistent improvement in the clinical longevity of adhesive restorations, and its benefits appear to depend on the adhesive strategy and application protocol [9,10], being effective against Gram-positive and Gram-negative bacteria [11]. Its efficacy against *Staphylococcus* species appears to be reduced [12]. At low concentrations, it increases cell wall permeability (bacteriostatic); at high concentrations, it precipitates cellular contents (bactericidal) [8,13]. In clinical practice, the preferred concentration of CHX is 2%, with a wide range of reported application times in the literature, 60 s being the most commonly reported duration [14]. At this concentration, it is considered biocompatible [15] and toxicologically safe [16].

H_2_O_2_ is a commonly used disinfectant in dental applications. It can be used for the disinfection of vital abutments, with a concentration of 3% (3% H_2_O_2_), as employed in previous prosthodontic investigations [17]. The antimicrobial effect is based on the oxidation of bacterial DNA, proteins, and lipids [18], which can compromise the immediate bond strength of resin cements [2,19].

In recent decades, photodynamic therapy (PDT), which involves the irradiation of photosensitizing agents with a diode laser, has been proposed as an alternative to conventional techniques, with increased effectiveness. PDT results in the generation of reactive oxygen species, leading to bacterial death on dentinal surfaces or in organized biofilms [20]. Alrahlah et al. demonstrated the effectiveness of photosensitizing therapy in dentin decontamination, which positively influenced the bond strength of self-etch adhesive resins [21]. 

*Streptococcus mutans* and *Lactobacilli* represent the classic cariogenic microorganisms in the oral cavity; however, *Staphylococcus aureus*, *C. albicans*, and other microorganisms can be identified in oral biofilms, different oral infections, and denture-induced stomatitis. Salivary pH and buffering capacity may influence their persistence and pathogenicity [22]. MRSA is clinically important in infection control. MRSA can colonize both the oral cavity and the surfaces of prosthetic restorations, making it a potential source of infection, especially because of its increased resistance to antimicrobial treatment [23,24]. Due to its adhesive capacity, ability to survive in harsh conditions, and capacity to form biofilms, MRSA is considered a relevant resistant Gram-positive model for testing the efficacy of different disinfection protocols [25,26].

*C. albicans* is one of the most common yeasts in the oral cavity and is involved in opportunistic infections, infections of the oral mucosa, denture-induced stomatitis, and dental biofilm-associated infections [27,28]. Its ability to penetrate dentinal tubules, its good adhesion to tooth structure or dental materials, and its interaction with other microorganisms contribute to the stabilization of microbial colonies and to biofilm formation on dentinal surfaces [14,29]. The elimination of a biofilm formed by *C. albicans* by conventional methods is challenging due to its increased tolerance to antimicrobial agents [30,31]. The effectiveness of disinfectants in reducing the microbial load on dentinal surfaces is variable and depends on the experimental conditions [2,32].

The proper selection of microorganisms in research studies examining the efficacy of disinfectants in dentistry is essential. In prosthodontics, eliminating microorganisms from the abutment surface is vital before the adhesive cementation of restorations. MRSA is considered a commonly used reference strain in research studies [33]. Its high survival rate and ability to form stable biofilms under unfavorable conditions [34] have made it the starting point for several studies investigating its resistance to antibiotic therapy, but its susceptibility to various antimicrobial disinfectants on dentinal surfaces has not been sufficiently investigated [35]. Although MRSA is not considered a typical organism of the oral environment, oral carriage and transient colonization have been reported in the literature, especially in susceptible patients and in healthcare settings. In this study, MRSA was included as a model of a resistant Gram-positive organism rather than as a representative of the dentinal microbiota. It is capable of adhering to surfaces, forming biofilms, and persisting under adverse environmental conditions. Hence, it can serve as an experimental model for comparing the antimicrobial efficacy of dentin disinfection protocols [23]. Most studies have evaluated the effectiveness of a limited number of disinfectants or microorganisms [2,36], while factors such as application time, penetration depth, and microorganism type have not been sufficiently investigated [32]. Comparative studies evaluating antimicrobial and chemo-mechanical approaches remain limited, highlighting the need for broader experimental designs [37]. Evidence regarding antimicrobial efficacy on prepared coronal dentin in a prosthodontic setting remains limited. The effectiveness of surface-disinfection protocols in prepared coronal dentin at different dentin depths has been poorly investigated. Therefore, the present in vitro study aimed to comparatively evaluate the efficacy of conventional chemical disinfection (2% CHX, 3% H_2_O_2_) and photodynamic therapy (PDT) with 1% toluidine blue (TB) against MRSA and *C. albicans* on prepared and experimentally contaminated human dentin, with the assessment of microbial recovery separately at predefined sampling sites in superficial and deep dentin. This depth-sensitive approach provides information on the extent to which the antimicrobial effect of surface disinfection extends beyond superficial dentin to its deeper layers. 

In addition, the penetration of disinfectants at different dentin depths was assessed indirectly by evaluating the microbial growth reduction after their application exclusively on the external dentin surface. The primary null hypothesis was that no significant differences would be observed among the tested disinfection protocols. The secondary hypothesis was that the antimicrobial efficacy of various dentin disinfection methods would vary according to microorganism type (bacteria and fungi), dentin depth and application time.

## 2. Materials and Methods

This in vitro study was approved by the Ethics Committee of the “George Emil Palade” University of Medicine, Pharmacy, Science and Technology of Târgu Mureș (protocol code 3723, approved on 27 March 2025).

### 2.1. Study Design, Sample Size

G*Power version 3.1.9.6 software (Franz Faul, Universität Kiel, Kiel, Germany) was used to calculate the sample size. Based on previous in vitro studies evaluating dentin disinfection protocols [38,39], an effect size of *w* = 0.40 was considered for χ^2^ tests, with a significance level of *α* = 0.05 and a statistical power of 80%. Considering six disinfection protocols and four ordinal microbial-score categories (df = 15), the minimum required sample size was estimated at 118 experimental units. The tooth was considered the experimental unit. A total of 144 teeth were included in the study, each providing paired measurements from superficial and deep dentin.

All the procedures were conducted at the Department of Fixed Prosthodontics of the Faculty of Dental Medicine, Applied Nanotechnology in Prosthodontics Research Laboratory, and the Department of Microbiology of the “George Emil Palade” University of Medicine, Pharmacy, Science, and Technology of Târgu Mureș.

The inclusion criteria for the selection of teeth included permanent teeth (molars) with a sound clinical crown (no caries, endodontic treatments, or restorations). The exclusion criteria included teeth with decay, direct or indirect restorations, deciduous teeth, and teeth not indicated for extraction. 

**Figure 1:**
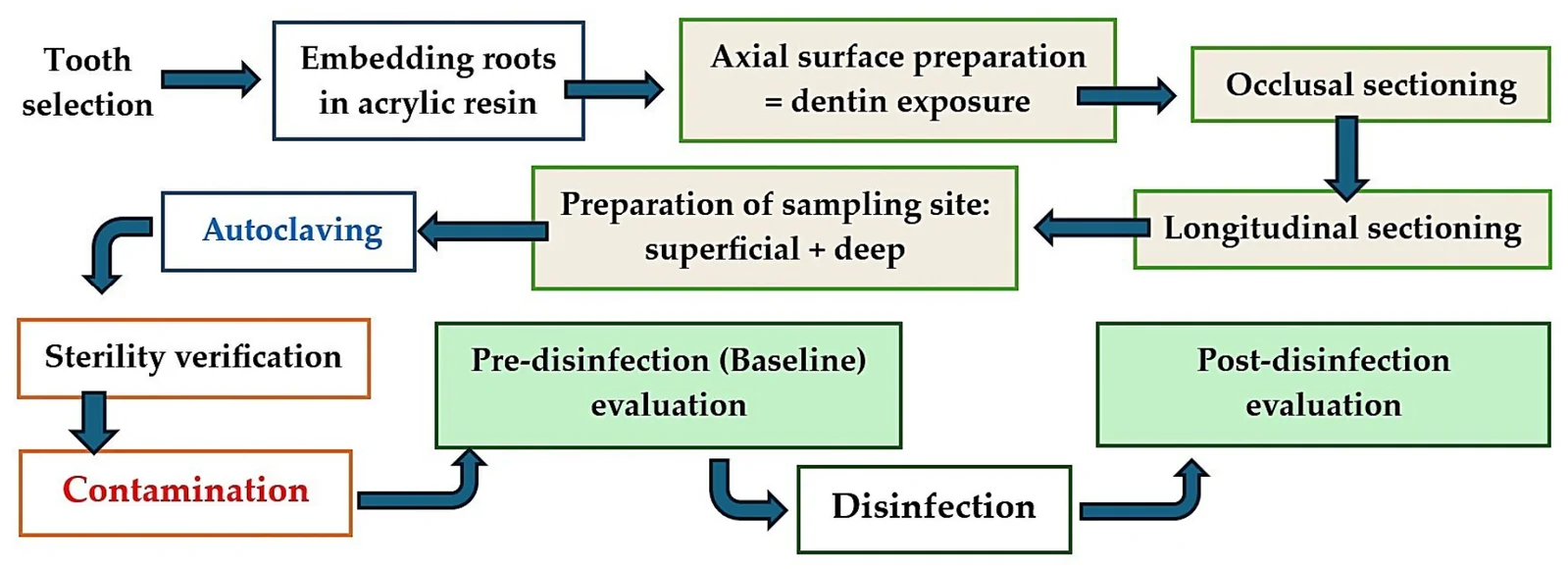
Schematic representation of the sequential stages of the experimental workflow.

The experimental workflow is summarized in Figure 1.

### 2.2. Minimum Inhibitory Concentration (MIC) Determination

Prior to the dentin experiments, a planktonic susceptibility assay was performed as a baseline control to verify the intrinsic antimicrobial activity of the tested disinfectants against both reference microorganisms under standardized and fully controlled conditions. The purpose of this preliminary assay was to confirm that the tested microorganisms were susceptible to the disinfectants under planktonic conditions, thereby providing a reference against which the reduced or variable antimicrobial effects observed in the more complex dentin environment could be interpreted.

**Figure 2:**
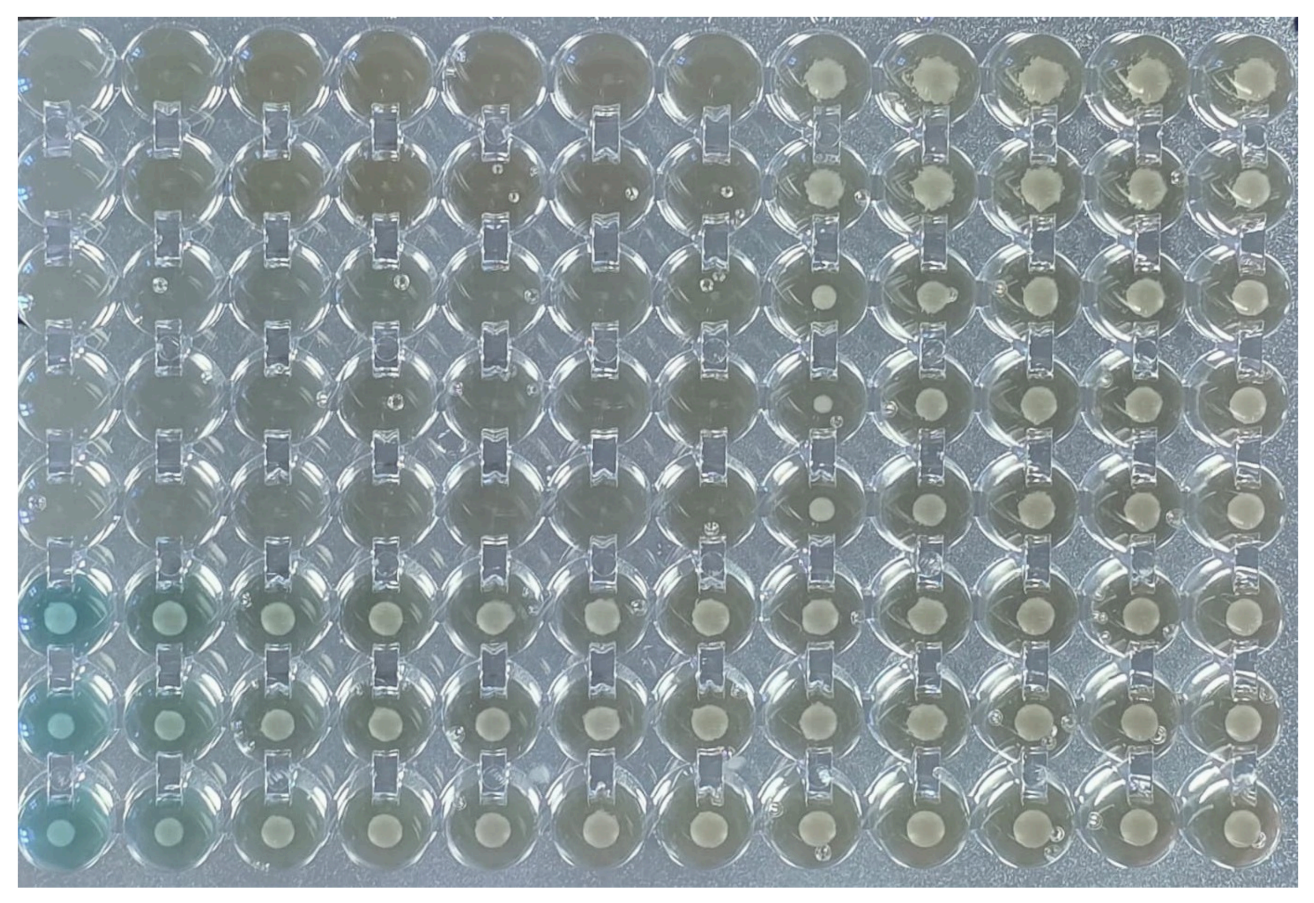
Representative 96-well microplate illustrating the visual assessment of microbial growth for MIC determination.

The antimicrobial activity of the disinfectants was evaluated after 24 h for bacteria and after 48 h for fungi using the microdilution method [40]. Twelve serial dilutions of each disinfectant were prepared (Figure 2), starting from a 50% concentration of each solution used in the experiment. Following incubation, microbial growth was assessed visually based on the presence or absence of turbidity [41]. The MIC was defined as the lowest tested disinfectant concentration showing no visible microbial growth (the last well without visible microbial growth). No subculturing of growth-negative wells was performed; therefore, minimum bactericidal concentration (MBC) and minimum fungicidal concentration (MFC) values were not determined. 

The antimicrobial activity of disinfectants was then evaluated in vitro on extracted posterior teeth, simulating a clinical scenario encountered during prosthodontic approaches involving tooth preparation for adhesively cemented indirect restorations.

### 2.3. Specimen Preparation

Organic residues on the surface of extracted teeth were removed by ultrasonic and manual scaling.

**Figure 3:**
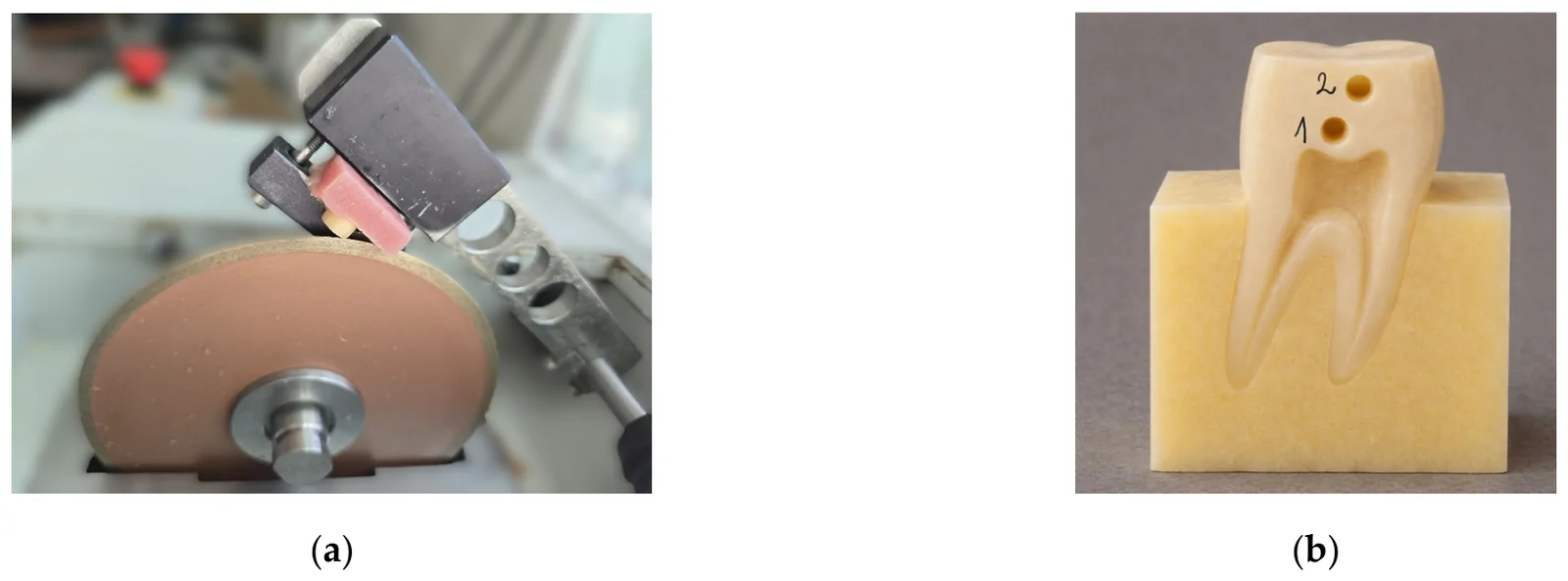
The preparation of the specimens: (**a**) sectioning the tooth embedded in an acrylic block with the Micracut 151 machine (Metkon Instruments Inc., Turkey); (**b**) schematic representation of the sampling sites on the longitudinal section: (1) deep sample site, (2) superficial sample site (AI-assisted model based on an original specimen image).

The preparation of the specimens started with embedding the cleaned roots of the selected teeth in self-curing acrylic resin (polymethyl methacrylate) and leaving the clinical crowns exposed. The axial walls of the crowns were prepared using a flame-shaped diamond bur in a high-speed handpiece under continuous water cooling until complete dentin exposure on all surfaces. The occlusal surface of each tooth was sectioned horizontally using the Micracut 151 sectioning device (Metkon Instruments Inc., Bursa, Turkey) at 500 RPM under continuous water-based cooling with METCOOL coolant (3% METCOOL, 97% water), followed by longitudinal sectioning of the tooth at the midpoint of the occlusal surface with the same protocol and sectioning device (Figure 3a). Following longitudinal sectioning, two standardized spherical sampling cavities were prepared on the exposed sectioned dentin surface of each specimen to standardize microbiological sample collection from different dentin depths. The cavities were positioned under loupe magnification at two predefined anatomical levels, corresponding to superficial and deep dentin, respectively. The first site, located in superficial dentin, was prepared with the closest edge approximately 0.5 mm below the occlusal surface, while the second site, corresponding to deep dentin, was prepared approximately 0.5 mm above the roof of the pulp chamber using a contra-angle handpiece (10,000 RPM) and a 2 mm diameter spherical diamond bur (Figure 3b). The bur was advanced to approximately half of its spherical diameter, corresponding to a preparation depth of approximately 1 mm. To ensure standardization, all procedures were performed by the same operator.

A schematic representation of the specimen with the sampling sites (Figure 3b) was generated by refining an original photograph using AI-assisted image processing.

The specimens obtained were autoclaved at 134 °C, without drying, to preserve the structural integrity of the dentin and avoid the potential collapse of the collagen network, which could influence microorganism and disinfectant penetration through dentin tubules. It should be noted that Pashley et al. reported no structural or permeability modifications following autoclaving of extracted teeth [42]. Following autoclaving, the teeth were stored in sterile distilled water, in a closed, sterile container, at room temperature (21 °C) until their use in the study. The distilled water was changed weekly. 

Prior to experimental contamination, samples of the storage medium were aseptically collected and cultured at 37 °C for 24 and 48 h to assess post-sterilization microbial contamination. No microbial growth was detected.

### 2.4. Surface Contamination

After preparation, the specimens were divided into two microbiological contamination groups based on the tested microorganism: *S. aureus* MRSA (ATCC 43300) and *C. albicans* (ATCC 10213). The microbial suspensions were prepared and used separately for each microorganism.

The specimens assigned to each group were immersed in a common container containing the nutrient broth with a standardized microbial suspension, without masking the sectioned surfaces and the sampling cavities. The microbial suspensions were adjusted densitometrically to 0.5 McFarland units (approximately 1.5 × 10^6^ CFU/mL for *S. aureus* and approximately 1.5 × 10^6^ CFU/mL for yeasts). The microbial strains were obtained from the Culture Collection of the Department of Microbiology at the “G. E. Palade” University of Medicine, Pharmacy, Science, and Technology of Târgu Mureș, Romania.

The purpose of this evaluation was to determine the antimicrobial efficacy after disinfectant penetration to different dentin depths. The samples were incubated for 24 and 48 h at 37 °C to allow microorganisms to grow. Following inoculation and prior to the application of the disinfection protocols, baseline microbial growth was assessed for each specimen by visual examination, based on colony density (abundant, moderate, scant colony growth, or absence of growth). This pre-disinfection assessment was performed to verify successful experimental contamination at the individual specimen level.

### 2.5. Sample Collection

The specimens were incubated in the microbial suspension. The sampling sites remained moist, allowing microbial material to be collected using sterile paper points. To assess the baseline microbial growth, one sterile paper point was placed in each sampling site for 10 s. A separate sterile paper point was used for the superficial and deep sampling sites. Each tooth provided two microbiological measurements, one from the superficial and another from the deep sampling site. Paper-point sampling was selected instead of rotary bur sampling to minimize mechanical disturbance and potential dispersion of the microbial suspension during sample collection. Following collection, each paper point was immediately brought into direct contact with the surface of the corresponding culture medium (mannitol salt agar for MRSA and Sabouraud agar for *C. albicans*) to transfer the collected microbial material. Post-disinfection microbiological samples were collected from the predefined sampling sites after a 60 or 120 s exposure time. No intermediate elution, neutralization, or vortexing step was performed. The same sample collection and inoculation procedures were applied to all pre- and post-disinfection samples, and all microbiological procedures were performed by the same operator. The Petri plates were incubated for 24 h for bacteria and 48 h for fungi at 37 °C under aerobic conditions. 

Due to the presence of confluent or semi-confluent microbial growth on several culture plates, an exact CFU count was not considered sufficiently reliable. Consequently, microbial growth was assessed using a semi-quantitative visual ordinal scoring system based on colony density and classified as follows: absent growth, scant growth, moderate growth, or abundant growth. This method provided an ordinal estimation of viable microbial growth rather than an exact CFU count. The scoring system was selected to compare relative microbial reduction across protocols under standardized experimental conditions. The same scoring criteria were applied to both the pre-disinfection (baseline) and post-disinfection assessments. The same operator, an experienced prosthodontic specialist, performed all procedures for specimen preparation and sample collection. The same experienced microbiology specialist performed all microbiological procedures (preparation of the microbial suspension, contamination of the specimens, inoculation, and microbial growth evaluation) to reduce operator-related variability and standardize methods. To assess intra-examiner reliability, a random subset of 34 measurements (12% of the total) was re-evaluated after 2 weeks. The agreement between the two assessments was measured using the weighted Cohen’s kappa coefficient, which showed very good agreement (κ = 0.87; *p* < 0.001). 

### 2.6. Surface Disinfection

Following contamination and incubation, the specimens from each microbial group were randomly assigned to six experimental subgroups in accordance with the disinfection protocol. In all cases, disinfectants were applied topically, exclusively to the external axial and occlusal dentin surfaces, without contact with the sectioned surfaces and sampling cavities. The application was performed by rubbing with a microbrush for either 30 or 60 s (application time). The antimicrobial efficacy of the disinfectants was evaluated by assessing microbial growth in samples collected after 60 and 120 s from the cavities following disinfectant penetration. 

The groups were divided based on experimental protocols as follows:

Group 1 (CHX_1): Disinfection with 2% CHX, applied to the external dentin surface by swabbing the surface with applicators for 30 s.

Group 2 (CHX_2): Disinfection with 2% CHX, applied to the external dentin surface by swabbing the surface with applicators for 60 s.

Group 3 (H_2_O_2__1): Disinfection with 3% H_2_O_2_, applied to the external dentin surface by swabbing the surface with applicators for 30 s.

Group 4 (H_2_O_2__2): Disinfection with 3% H_2_O_2_, applied to the external dentin surface by swabbing the surface with applicators for 60 s.

Group 5 (PDT_1): Disinfection with a diode laser via PDT with 1% TB applied to the external dentin surface by swabbing the surface with applicators for 30 s. After the application of TB, the specimens were stored in the dark for 1 min, after which the dentin surfaces were irradiated with the Lasotronix Smart M Pro diode laser (Lasotronix Sp. z o.o., Piaseczno, Poland) for 30 s at 1 mm, in pulse mode, with a power of 1.5 W at 635 nm.

Group 6 (PDT_2): Disinfection with a diode laser via PDT with 1% TB applied to the external dentin surface by swabbing the surface with applicators for 60 s. After the application of TB, the specimens were stored in the dark for 1 min, after which the dentin surfaces were irradiated with the Lasotronix Smart M Pro diode laser (Lasotronix Sp. z o.o., Piaseczno, Poland) for 30 s at 1 mm, in pulse mode, with a power of 1.5 W at 635 nm.

The database included 288 valid measurements, evenly distributed across the six experimental protocols (CHX_1, CHX_2, H_2_O_2__1, H_2_O_2__2, PDT_1, and PDT_2), the two microorganisms (MRSA and *C. albicans*), evaluation times after disinfection (60 s and 120 s), and dentin depths (superficial and deep), with six measurements for each combination of protocol–microorganism–time–depth. Two independent time-related variables were evaluated. For each application-time condition (30 or 60 s), post-disinfection microbiological sampling was performed after a waiting period of either 60 or 120 s (sampling time). No additional intervention was performed between disinfectant application and microbiological sampling.

### 2.7. Statistical Analysis

Statistical analysis was conducted using GraphPad Prism 11 for macOS, version 11.0.0 (93), (GraphPad Software, Boston, MA, USA) and Python, version 3.14.7 (Python Software Foundation, Wilmington, DE, USA). The significance threshold was set at *p* < 0.05, and all tests were two-tailed. Because the outcome variable was ordinal, data were described using absolute frequencies, percentages, median (Med), and interquartile range (IQR). Mean (M) and standard deviation (SD) were also reported to provide an overall descriptive summary.

The tooth was considered the experimental unit (*n* = 144). Each tooth provided two microbiological measurements, corresponding to superficial and deep dentin, resulting in a total of 288 measurements. Because the two measurements obtained from the same tooth were not statistically independent, within-tooth correlation was taken into account in the inferential analysis.

Ordinal generalized estimating equations (GEE) with a cumulative logit link were used to evaluate the effects of disinfection protocol, microorganism, sampling time, and dentin depth on microbial scores while accounting for the paired data structure. Tooth identity was specified as the clustering variable, using an exchangeable working correlation structure and robust standard errors. PDT_1 was used as the reference category for the disinfection protocol, *C. albicans* for microorganism, 120 s for sampling time, and deep dentin for dentin depth. Results were expressed as odds ratios (ORs), 95% confidence intervals (CIs), and corresponding *p*-values.

The overall effects of the disinfection protocol and the other predictors were evaluated using Wald χ^2^ tests derived from the GEE models. Separate models were also constructed for MRSA and *C. albicans*.

For comparisons stratified simultaneously by sampling time and dentin depth, each tooth contributed only one measurement to a given analysis. Therefore, the Kruskal–Wallis test was used to compare microbial scores among disinfection protocols within these strata. Effect sizes were expressed as epsilon squared (ε^2^).

When a significant overall protocol effect was identified, pairwise comparisons among disinfection protocols were performed within the GEE framework, with *p*-values adjusted for multiple comparisons using the Holm method. In this model, higher microbial scores indicated greater residual microbial growth. Therefore, odds ratios below 1 indicated lower odds of belonging to higher microbial-score categories and, consequently, greater antimicrobial effectiveness relative to the reference category. Conversely, odds ratios above 1 indicated higher odds of belonging to higher microbial-score categories and greater residual microbial growth relative to the reference category.

## 3. Results

### 3.1. Determination of Minimum Inhibitory Concentrations (MICs)

The MICs obtained for the tested disinfectants differed for both *S. aureus* and *C. albicans* (Table 1).

H_2_O_2_ demonstrated the most effective antibacterial effect against *S. aureus*, even at the highest tested dilution (0.0007%). TB alone showed weak antibacterial activity, whereas when combined with PDT, no inhibitory effect against MRSA was observed under the tested conditions.

CHX exhibited the strongest antifungal activity against *C. albicans*, with the lowest MIC value of 0.0009%. H_2_O_2_ showed moderate antifungal activity, whereas TB, when combined with PDT, did not exhibit measurable inhibitory activity against *C. albicans*.

None of the disinfectants used demonstrated complete antifungal activity at any of the 12 serial dilutions against *C. albicans*.

### 3.2. Descriptive Statistics

A total of 144 teeth were included in the final analysis, providing 288 paired microbiological measurements from superficial and deep dentin. The samples were distributed across six disinfection protocols (CHX_1, CHX_2, H_2_O_2__1, H_2_O_2__2, PDT_1, and PDT_2) and were evaluated for MRSA and *C. albicans* at 60 and 120 s.

**Figure 4:**
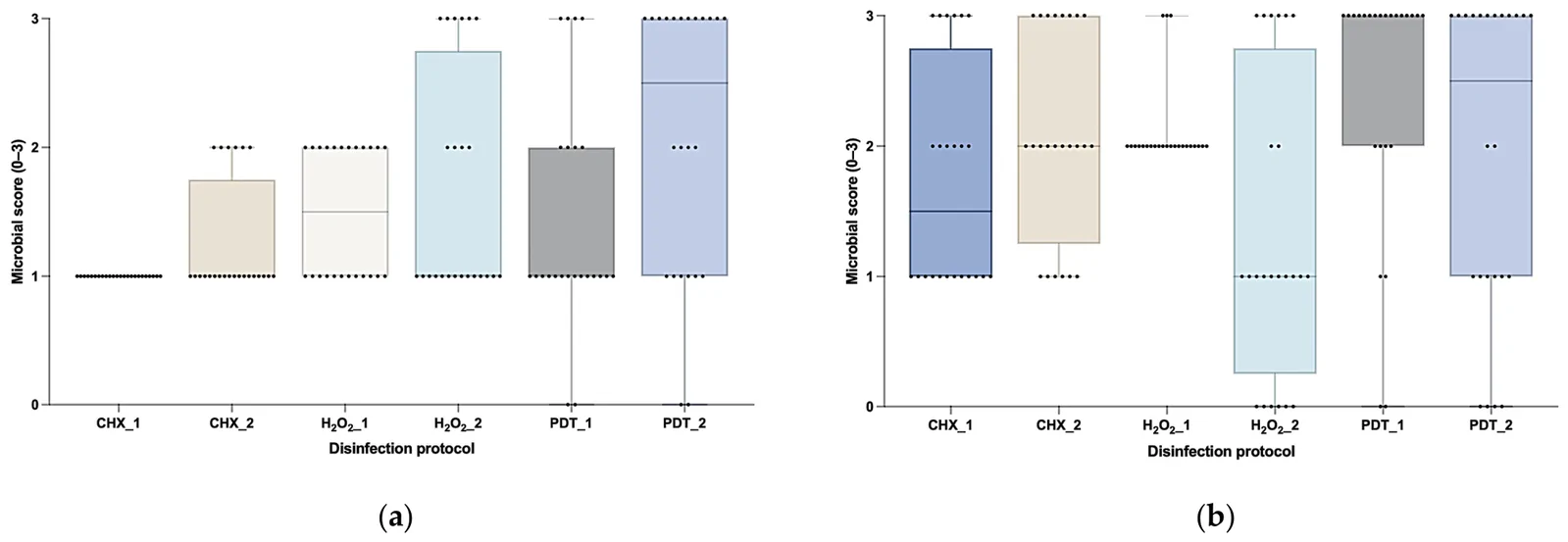
Distribution of microbial scores across disinfection protocols: (**a**) MRSA; (**b**) *C. albicans*. Boxplots represent the median and interquartile range, with whiskers indicating minimum and maximum values. Individual data points are displayed.

The distribution of microbial scores varied among the disinfection protocols for each microorganism used in this study (Table 2). For MRSA, CHX-based protocols showed the lowest scores, especially in the CHX_1 group (Med = 1.0, IQR: 1.0–1.0), whereas the PDT_2 protocol had the highest scores (Med = 2.5, IQR: 1.0–3.0) (Figure 4a). Complete absence of detectable MRSA growth was observed only with the PDT_1 and PDT_2 protocols (8.3%). For *C. albicans*, the lowest microbial scores were observed in the H_2_O_2__2 group (Med = 1.0, IQR: 0.75–2.25), followed by CHX_1, while the PDT_1 protocol showed the highest scores (Figure 4b). Complete microbial eradication (score 0) was observed in three of the six protocols, with the highest frequency observed for H_2_O_2__2 (Table 2). 

### 3.3. Comparisons Among Disinfection Protocols

The overall analysis showed significant differences in microbial scores among the six disinfection protocols for both MRSA and *C. albicans* (Table 3). Sampling time was also significantly associated with microbial scores for both microorganisms, whereas dentin depth showed no significant effect. In the combined analysis, disinfection protocol, microorganism, and sampling time were significant predictors of microbial scores, while dentin depth was not.

### 3.4. Stratified Analysis According to Sampling Time and Dentin Depth

To observe the effects of disinfection protocols, analyses were stratified by time (60 s vs. 120 s) and dentin depth (superficial vs. deep) using the Kruskal–Wallis test. For MRSA, significant differences were observed among disinfection protocols at both dentin depths at 60 s and in deep dentin at 120 s, while no significant differences were found in superficial dentin at 120 s. For *C. albicans*, significant differences were observed among protocols in deep dentin at both sampling times, while no significant differences were found in superficial dentin (Table 4). 

### 3.5. Factors Associated with Microbial Scores

**Figure 5:**
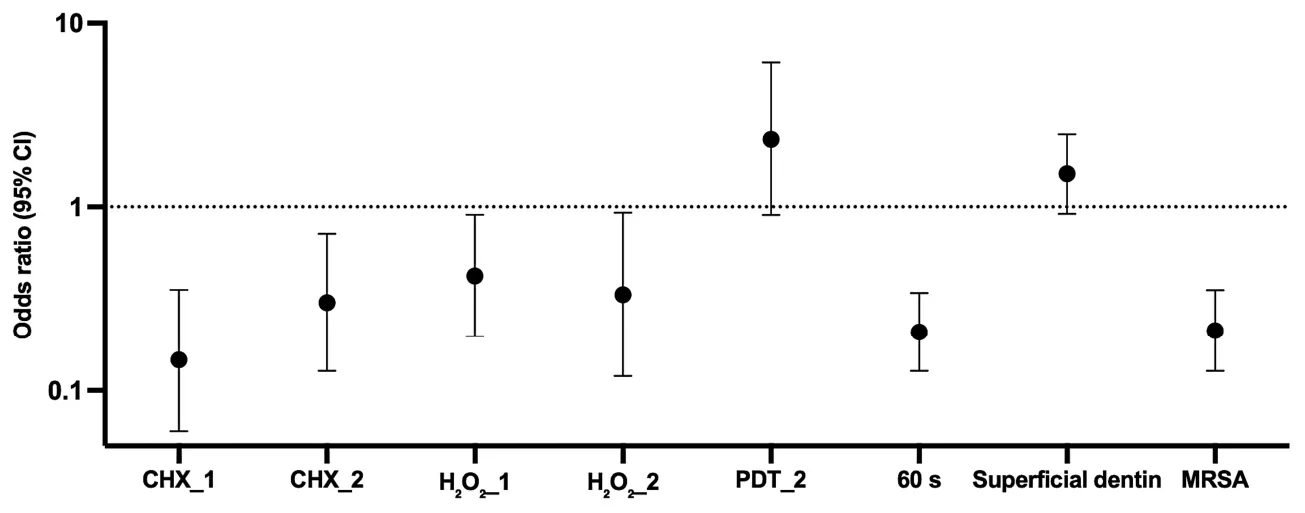
Adjusted odds ratios (ORs) and 95% confidence intervals derived from the overall ordinal generalized estimating equation (GEE) model. The dotted line represents the null value (OR = 1). Reference categories were PDT_1 for the disinfection protocol, 120 s for application time, deep dentin for dentin depth, and *C. albicans* for the microorganism.

Ordinal GEE models were used to evaluate associations among disinfection protocol, microorganism, sampling time, dentin depth, and microbial score while accounting for the paired measurements obtained from the same tooth (Table 5). Protocol-related associations differed for MRSA and *C. albicans*, while sampling time showed a consistent significant association with microbial scores. Dentin depth was not significantly associated with microbial score in either the microorganism-specific or overall models (Figure 5).

### 3.6. Post Hoc Comparisons 

Post hoc pairwise comparisons were performed using the Holm adjustment for multiple comparisons. For MRSA, statistically significant differences were observed among CHX_1 and H_2_O_2__1 (*p* < 0.001), CHX_1 and H_2_O_2__2 (*p* = 0.004), CHX_1 and PDT_2 (*p* < 0.001), CHX_2 and PDT_2 (p < 0.001), H_2_O_2__1 and PDT_2 (*p* < 0.001), and PDT_1 and PDT_2 (*p* = 0.005).

For *C. albicans*, statistically significant differences were observed among PDT_1 and CHX_1 (*p* = 0.019), PDT_1 and H_2_O_2__2 (*p* = 0.003), and H_2_O_2__2 and PDT_2 (*p* = 0.031). No other pairwise comparisons were statistically significant.

## 4. Discussion

In the present study, the evaluated dentin disinfection protocols differed in antimicrobial efficacy, with distinct response patterns for MRSA and *C. albicans*. These findings underscore the importance of considering both the disinfectant agent and the target microorganism when evaluating dentin disinfection strategies.

The use of MRSA strains in experimental studies is common, making them a relevant basis for evaluating the efficacy of antimicrobial disinfectants against resistant bacteria [23,25,43]. Investigating the effectiveness of disinfectants against MRSA can provide relevant information on their antimicrobial activity and their capacity to reduce the resistant microbial load in the oral biofilm, as MRSA can form biofilms and colonize the oral cavity [44].

MRSA and *C. albicans* strains used in this study allow evaluation of the efficacy of dentin disinfection methods against representative microorganisms (bacteria and fungi), providing a complex model of the antimicrobial potential of the tested solutions [28,45,46].

According to the literature, the complete elimination of microorganisms from dentin surfaces represents a major challenge [32,34,47,48,49,50]. The results of the present study are consistent with previous studies showing that the antimicrobial efficacy of dentin disinfection protocols may vary depending on the microorganism tested [28,34] and experimental conditions, including application time and dentin depth [2,18,32,47]. Complete microbial eradication was less common among the protocols evaluated. This can be explained by the irregular dentinal tubular structure, which provides protective niches for microorganisms and makes it difficult for surface-applied disinfectants to penetrate [48,49]. Thus, complete eradication of microorganisms is difficult to achieve, as our results confirmed. Score 0 occurred infrequently across most protocols, although it was more frequently observed for H_2_O_2__2 against *C. albicans*. This finding may be related to the oxidative antimicrobial mechanism of H_2_O_2_ [18,51,52,53].

Differences among disinfection protocols were significant for both microorganisms, although the response patterns differed for MRSA and *C. albicans*. The effect sizes observed in the stratified analyses indicate that, under several experimental conditions, the differences among protocols were substantial and not limited to statistical significance alone. This variable response may be related to the complex structure of dentin and the smear layer resulting from tooth preparation [54]. The tubular dentin structure favors microbial penetration and colonization, allowing microorganisms to survive in deeper dentin layers and remain protected against surface-applied antimicrobial solutions [55]. Several studies have demonstrated a 200–300 μm penetration depth for microorganisms into dentinal tubules, where they are protected from antimicrobial agents [48,55,56,57]. Compared with the exposed dentin surface, deeper dentinal regions may provide a more confined microenvironment characterized by restricted nutrient diffusion and potentially reduced oxygen availability. *C. albicans* is metabolically flexible and capable of adapting to both normoxic and hypoxic environments, while environmental cues can also influence its yeast-to-hypha transition and biofilm phenotype [58,59].

Hyphal growth and biofilm organization may facilitate persistence within the dentinal environment and contribute to reduced susceptibility to antimicrobial treatment [60]. Therefore, the different microbial responses observed between superficial and deep dentin may partly reflect depth-dependent microenvironmental conditions. However, oxygen concentration and fungal morphology were not directly assessed in the present study; consequently, this explanation remains mechanistic and should be interpreted with caution.

In our study, chemical disinfection generally showed a more consistent antimicrobial effect against the tested microorganisms than PDT, although the response depended on the microorganism and protocol evaluated. This pattern was further supported by the ordinal GEE analysis, which accounted for the paired superficial and deep dentin measurements obtained from the same tooth. In the overall model, CHX_1, CHX_2, H_2_O_2__1, and H_2_O_2__2 were significantly associated with lower odds of higher microbial scores compared with PDT_1, whereas PDT_2 did not differ significantly from the reference protocol. However, microorganism-specific analyses revealed different response patterns, indicating that the relative effectiveness of the protocols was not uniform for MRSA and *C. albicans*. These findings are consistent with previous studies showing that antimicrobial performance depends on both the disinfecting agent and the characteristics of the target microorganism [33,61,62,63]. 

For MRSA, 2% CHX (applied for 30 s – CHX_1) demonstrated the most favorable antimicrobial effect, with lower microbial scores compared with the other evaluated protocols. This finding was supported by the MRSA-specific GEE model, in which CHX_1 was associated with significantly lower odds of higher microbial scores compared with PDT_1. In contrast, CHX_2 did not differ significantly from the reference protocol. A cationic mechanism allows CHX to disrupt cellular homeostasis by interacting with and penetrating the bacterial membrane. These findings support the role of CHX as an effective dentin disinfectant reported in several studies [4,11]. Besides its antimicrobial effect, CHX protects the collagen fibers in the hybrid layer against matrix metalloproteinases (MMPs), contributing to the long-term stability of the adhesive interface and bond strength [2,64]. From a clinical perspective, the objective of disinfection is not only to reduce microbial load but also to preserve adhesive performance to dentin, particularly in clinical situations where reduced enamel thickness or structural loss may influence adhesive protocols over time [65].

However, 3% H_2_O_2_ applied for 60 s (H_2_O_2__2) demonstrated a particularly favorable antimicrobial effect against *C. albicans*, with the highest frequency of complete microbial eradication. This is supported by the *C. albicans*-specific GEE model, in which both H_2_O_2_ protocols were associated with significantly lower odds of higher microbial scores compared with PDT_1, with the strongest association observed for H_2_O_2__2. In contrast, neither H_2_O_2_ protocol showed a significant association with microbial score in the MRSA-specific model, suggesting a microorganism-dependent response under the conditions evaluated in this study. These results, supported by the literature [18], may be related to the oxidative mechanisms of H_2_O_2_, which can damage fungal membranes and other cellular components [51,52,53]. These findings are consistent with studies describing the response of fungal cells to oxidative stress [51,66,67]. MIC test results were not fully consistent with those observed at the dentin level. These differences can be attributed to the fact that MIC tests are conducted under controlled conditions, whereas dentin’s complex structure may limit contact between disinfectants and microorganisms [1,2]. Therefore, MIC test results cannot be directly extrapolated to dentin, where the antimicrobial effect may be influenced by the dentinal substrate and the limited penetration of the agent into the dentinal tubules [2].

Overall, the tested disinfectants showed a less favorable antimicrobial response against *C. albicans* than against MRSA. This pattern was supported by the overall GEE model, in which the microorganism was significantly associated with microbial score. The ability of *C. albicans* to form biofilms and colonize dentinal tubules [28,30] may contribute to its persistence within the dentinal substrate. Differences in dentinal tubule density and diameter may also influence the penetration of antimicrobial agents [49,68]. Therefore, chemical disinfection should complement the mechanical removal of infected dentin, particularly in areas where microorganisms may persist within the dentinal tubules [63,69].

The antimicrobial performance of PDT observed in this study was variable and depended on the microorganism and protocol evaluated. In particular, the MRSA-specific GEE model showed that PDT_2 was associated with significantly higher odds of higher microbial scores compared with PDT_1, whereas no significant difference between the two PDT protocols was observed for *C. albicans* or in the overall model. This variability may be related to the limited penetration of the photosensitizer and light energy into dentinal tubules and to the dependence of PDT on several parameters, including photosensitizer concentration, irradiation time, wavelength, power output, and light delivery, which can influence photodynamic antimicrobial activity [70,71]. These findings are consistent with previous studies demonstrating variability in the antimicrobial performance of PDT under different experimental conditions and against different microorganisms. While Tisler et al. and Alrahlah et al. described the efficacy of PDT in reducing dental biofilms [20,21], in our experiment, 1% TB and PDT did not demonstrate a significant antimicrobial effect in MIC tests. Because surface or intradentinal temperature changes were not monitored during laser irradiation, a possible thermal contribution cannot be completely excluded. According to Harorli et al., laser irradiation can induce different biological responses depending on the parameters used [72]. Therefore, the PDT results should be interpreted as the outcome of the tested laser-assisted disinfection protocol under the present experimental parameters, and the relative contribution of photodynamic and possible thermal effects cannot be clearly distinguished. The literature suggests that the effectiveness of PDT depends on several factors, including photosensitizer penetration into the biofilm and the distribution of light energy, which may explain the variability of results among studies [32,73,74].

Stratified analysis revealed that differences among disinfection protocols varied according to sampling time and dentin depth. However, in the GEE models, sampling time was significantly associated with the microbial score, whereas dentin depth was not an independent significant predictor. Overall, 60 s was associated with lower odds of higher microbial scores compared with 120 s. Although microbial scores tended to be more favorable in superficial dentin under some experimental conditions, this pattern was not independently significant in the GEE analysis. The persistence of microorganisms within dentin may nevertheless be related to the complexity of the dentinal structure, as dentinal tubules can provide protected sites that limit exposure to disinfecting agents. Since in the present study the antimicrobial agents were applied to the coronally exposed dentin, their effect at deeper levels depends on penetration into the dentinal substrate [1,2]. Increasing application time did not improve antimicrobial efficacy, suggesting that its effect might depend on the antimicrobial agent and protocol used.

This experimental approach was designed to reproduce the clinical direction of disinfectant application, in which antimicrobial agents are applied to the prepared external dentin surface of the abutment. The standardized cavities on the sectioned surface were used only for sample collection, allowing the evaluation of residual microbial growth at superficial and deep dentin levels. Although this in vitro model cannot reproduce all clinical variables, it allowed standardized assessment of the antimicrobial effect from the treated external dentin surface toward deeper dentin levels.

Clinically, the results of this study suggest that CHX applied for short periods (30 s) can achieve effective bacterial control on prepared dentin. H_2_O_2_ may be useful when fungal contamination is suspected. These microorganism-specific responses suggest that the effectiveness of dentin disinfection may depend on both the antimicrobial agent and the microbial contamination involved. However, given the in vitro design of the present study, these findings should not be directly extrapolated to clinical conditions and require confirmation in further in vivo and clinical studies. Dentin disinfection should therefore be considered a complementary procedure aimed at reducing residual microbial contamination rather than achieving complete sterilization.

Our results need to be interpreted in the context of an in vitro experiment, with its limitations. The semi-quantitative visual ordinal scoring system does not provide precise numerical CFU counts (CFU/mL). The confluent or semi-confluent microbial colonies prevented accurate and reproducible colony counting. Although this approach allowed comparison among experimental groups, future studies should use serial dilutions, digital image analysis, or molecular methods to obtain more detailed quantitative microbial data. Microbial material was collected using paper points and transferred directly to the agar surface rather than being eluted into a defined volume and quantitatively plated. Variability in microbial transfer from the absorbent paper points cannot be excluded, and the resulting growth scores should be interpreted as semi-quantitative rather than absolute measures of the viable microbial load. The microorganisms used in this experimental model were mono-specific (MRSA and *C. albicans*), while in vivo conditions involve complex, polymicrobial biofilms with multiple microbial interactions. Furthermore, although MRSA provided a resistant Gram-positive challenge organism, it has limited representativeness of the microbial communities typically encountered on prepared dentin. Therefore, the clinical relevance and generalizability of the MRSA-related findings should be interpreted with caution [23]. Despite verification of sterility and successful experimental contamination, the absence of dedicated untreated, contaminated, saline-treated, and sterile control groups represents a limitation and restricts the distinction between mechanical and antimicrobial effects. In vitro conditions cannot reproduce salivary flow, mature biofilm formation (with possible resistance against antimicrobial agents), or the influence of mechanical factors. The absence of dentinal fluids and osmosis can influence the penetration of disinfectants within dentin, which was not directly measured in this study. The semi-quantitative evaluation of microbial growth in the sampling cavities provides indirect evidence of the antimicrobial activity achieved after the disinfectant penetrates the dentin. Future studies are necessary to evaluate the penetration of disinfectants into dentin. The absence of agent-specific chemical neutralization before post-disinfection microbiological sampling may result in continued antimicrobial activity after sampling (carryover effect) and overestimation of the observed antimicrobial effect. Future studies should incorporate validated agent-specific neutralization protocols prior to microbiological recovery. Although all microbiological procedures and evaluations were performed by the same operator to ensure procedural consistency, knowledge of treatment-group allocation may have introduced observer bias, particularly because microbial growth was assessed using a visual ordinal scoring system.

Despite the limitations, the findings of this research contribute to a better understanding of disinfectant protocols and the optimization of protocol selection and surface treatment to achieve optimal outcomes for adhesive cementation in modern prosthodontics. Literature data on this subject remain limited and scattered. 

Further in vivo studies are needed to evaluate the effectiveness of the tested protocols under polymicrobial biofilm conditions. Microbial interactions and potential synergistic effects of disinfectant combinations may modify the findings. Optimizing PDT parameters is essential. The impact of these protocols on shear bond strength, with in vivo validation of the results, needs to be evaluated.

From a clinical perspective, the development of standardized disinfection protocols for adhesive cementation would serve as a guide for practitioners to improve their clinical performance.

## 5. Conclusions

Within the limitations of this in vitro study, the dentin disinfection protocols evaluated demonstrated differential antimicrobial effectiveness, influenced by multiple factors, including the target microorganism, application time, dentin depth and the interval between application and microbiological sampling. Differences among disinfection protocols were observed for both microorganisms; however, these variations were not consistent across all experimental conditions, particularly for *C. albicans* in deep dentin and at 120 s. None of the evaluated protocols achieved complete microbial elimination, especially in deep dentin layers. The lowest MRSA microbial score was obtained with 2% CHX applied for 30 s, while for *C. albicans*, the most effective protocol was 3% H_2_O_2_ applied for 60 s. PDT showed lower and less consistent effects compared to conventional chemical disinfectants.

These findings suggest that the dentin disinfection protocol may vary according to the biological and anatomical conditions involved. The present findings were obtained under controlled, in vitro conditions. Direct translation into clinical recommendations is not warranted. Further studies using clinically representative models and in vivo designs are needed to determine the most effective and biologically appropriate dentin disinfection protocols.

## Figures and Tables

**Table 1:** MICs of the tested disinfectants against MRSA and *C. albicans*.

Disinfectants	Starting Concentration (%)	MIC for *C. albicans* (%)/48 h	MIC for MRSA (%) /24 h
H_2_O_2_	3	0.02	≤0.0007
CHX	2	≤0.0009	No inhibition observed
TB	1	No inhibition observed	0.25, 0.50, 0.0625
PDT	-	No inhibition observed	No inhibition observed

“-” not applicable; H_2_O_2_, hydrogen peroxide; CHX, chlorhexidine digluconate; TB, toluidine blue; PDT, photodynamic therapy; MIC, minimum inhibitory concentration; MRSA, methicillin-resistant *Staphylococcus aureus*.

**Table 2:** Descriptive statistics of microbial scores for each disinfection protocol.

Protocol	MRSA Median [IQR]	MRSA Mean ± SD	MRSA Score 0, n (%)	*C. albicans* Median [IQR]	*C. albicans* Mean ± SD	*C. albicans* Score 0, n (%)
**CHX_1**	1.0 [1.0–1.0]	1.00 ± 0.00	0 (0.0)	1.5 [1.0–2.25]	1.75 ± 0.85	0 (0.0)
**CHX_2**	1.0 [1.0–1.25]	1.25 ± 0.44	0 (0.0)	2.0 [1.75–3.0]	2.08 ± 0.78	0 (0.0)
**H_2_O_2__1**	1.5 [1.0–2.0]	1.50 ± 0.51	0 (0.0)	2.0 [2.0–2.0]	2.12 ± 0.34	0 (0.0)
**H_2_O_2__2**	1.0 [1.0–2.25]	1.67 ± 0.87	0 (0.0)	1.0 [0.75–2.25]	1.33 ± 1.13	6 (25.0)
**PDT_1**	1.0 [1.0–2.0]	1.42 ± 0.88	2 (8.3)	3.0 [2.0–3.0]	2.42 ± 0.97	2 (8.3)
**PDT_2**	2.5 [1.0–3.0]	2.08 ± 1.06	2 (8.3)	2.5 [1.0–3.0]	1.92 ± 1.21	4 (16.7)

H_2_O_2_, hydrogen peroxide; CHX, chlorhexidine digluconate; PDT, photodynamic therapy; MRSA, methicillin-resistant *Staphylococcus aureus*; IQR, interquartile range; SD, standard deviation.

**Table 3:** Overall effects of the disinfection protocol and other predictors on microbial scores.

Model	Predictor	Wald χ^2^	df	*p*-Value
**MRSA**	Protocol	62.98	5	<0.001
	Sampling time	15.13	1	<0.001
	Dentin depth	0.52	1	0.469
***C. albicans***	Protocol	18.77	5	0.002
	Sampling time	13.23	1	<0.001
	Dentin depth	2.45	1	0.117
**Overall**	Protocol	47.23	5	<0.001
	Microorganism	35.66	1	<0.001
	Sampling time	39.24	1	<0.001
	Dentin depth	2.61	1	0.106

MRSA, methicillin-resistant *Staphylococcus aureus*; df, degrees of freedom. Wald χ^2^ statistics were obtained from ordinal GEE models accounting for paired superficial and deep dentin measurements within each tooth. Statistical significance was set at *p* < 0.05.

**Table 4:** Comparison of microbial scores among disinfection protocols stratified by sampling time and dentin depth.

Microorganism	Time	Dentin Depth	H	df	*p*-Value	ε^2^
**MRSA**	60 s	Superficial	23.19	5	<0.001	0.606
		Deep	16.33		0.006	0.378
	120 s	Superficial	6.58		0.254	0.053
		Deep	21.88		<0.001	0.563
***C. albicans***	60 s	Superficial	10.63		0.059	0.188
		Deep	22.20		<0.001	0.573
	120 s	Superficial	7.95		0.159	0.098
		Deep	17.07		0.004	0.402

MRSA, methicillin-resistant *Staphylococcus aureus*; H, Kruskal–Wallis test statistic; df, degrees of freedom; ε^2^, epsilon-squared effect size. Comparisons were performed separately for each microorganism, sampling time, and dentin depth. Within each time–depth stratum, each tooth contributed a single measurement. Statistical significance was set at *p* < 0.05.

**Table 5:** Ordinal GEE analysis of factors associated with microbial scores.

Variable	MRSA OR (95% CI)	*p*-Value	*C. albicans* OR (95% CI)	*p*-Value	Overall OR (95% CI)	*p*-Value
**Protocol (reference: PDT_1)**						
**CHX_1**	0.121 (0.025–0.592)	0.009	0.116 (0.031–0.433)	0.001	0.147 (0.060–0.355)	<0.001
**CHX_2**	0.426 (0.109–1.656)	0.218	0.226 (0.058–0.879)	0.032	0.302 (0.128–0.714)	0.006
**H_2_O_2__1**	1.010 (0.312-3.268)	0.987	0.241 (0.075-0.771)	0.017	0.423 (0.198-0.906)	0.027
**H_2_O_2__2**	1.610 (0.361–7.190)	0.532	0.054 (0.012–0.252)	<0.001	0.334 (0.120–0.930)	0.036
**PDT_2**	8.112 (2.477–26.569)	<0.001	0.323 (0.097–1.072)	0.065	2.354 (0.903–6.136)	0.080
**Time (reference: 120 s)**						
**60 s**	0.220 (0.103–0.472)	<0.001	0.320 (0.173–0.591)	<0.001	0.209 (0.128–0.341)	<0.001
**Dentin depth (reference: deep dentin)**						
**Superficial**	1.307 (0.633–2.697)	0.469	1.789 (0.864–3.705)	0.117	1.515 (0.915–2.506)	0.106
**Microorganism (reference: *C. albicans*)**						
**MRSA**	-	-	-	-	0.213 (0.128–0.353)	<0.001

“-” not estimated because microorganism was not included as a predictor in the microorganism-specific models; H_2_O_2_, hydrogen peroxide; CHX, chlorhexidine digluconate; PDT, photodynamic therapy; MRSA, methicillin-resistant *Staphylococcus aureus*. OR, odds ratio; CI, confidence interval. Statistical significance was set at *p* < 0.05.

## Data Availability

The original contributions presented in this study are included in the article. Further inquiries can be directed to the corresponding author.

## Abbreviations

The following abbreviations are used in this manuscript:

CHX	Chlorhexidine digluconate
H_2_O_2_	Hydrogen peroxide
PDT	Photodynamic therapy
MRSA	Methicillin-resistant *Staphylococcus aureus*
TB	Toluidine blue
MIC	Minimum inhibitory concentration
CFU	Colony-forming unit
Med	Median
IQR	Interquartile range
M	Mean
SD	Standard deviation
OR	Odds ratio
CI	Confidence interval
RPM	Rotation/minute
ε^2^	Epsilon-squared effect size
df	Degrees of freedom
H	Kruskal–Wallis H statistic
MBC	Minimum bactericidal concentration
MFC	Minimum fungicidal concentration
GEE	Generalized estimating equation
